# Soluble oligomeric amyloid-β induces calcium dyshomeostasis that precedes synapse loss in the living mouse brain

**DOI:** 10.1186/s13024-017-0169-9

**Published:** 2017-03-21

**Authors:** Michal Arbel-Ornath, Eloise Hudry, Josiah R. Boivin, Tadafumi Hashimoto, Shuko Takeda, Kishore V. Kuchibhotla, Steven Hou, Carli R. Lattarulo, Arianna M. Belcher, Naomi Shakerdge, Pariss B. Trujillo, Alona Muzikansky, Rebecca A. Betensky, Bradley T. Hyman, Brian J. Bacskai

**Affiliations:** 1 Alzheimer Research Unit, Department of Neurology, Massachusetts General Hospital and Harvard Medical School, 114, 16th St., Charlestown, MA 02129 USA; 2 Department of Neuropathology, The University of Tokyo, Tokyo, Japan; 3 Skirball Institute, NYU School of Medicine, New York, NY 10016 USA; 4 Department of Biostatistics, Harvard School of Public Health, 50 Staniford Street, Boston, MA USA

**Keywords:** Alzheimer’s disease, Amyloid β oligomers, Calcium, In vivo imaging

## Abstract

**Background:**

Amyloid-β oligomers (oAβ) are thought to mediate neurotoxicity in Alzheimer’s disease (AD), and previous studies in AD transgenic mice suggest that calcium dysregulation may contribute to these pathological effects. Even though AD mouse models remain a valuable resource to investigate amyloid neurotoxicity, the concomitant presence of soluble Aβ species, fibrillar Aβ, and fragments of amyloid precursor protein (APP) complicate the interpretation of the phenotypes.

**Method:**

To explore the specific contribution of soluble oligomeric Aβ (oAβ) to calcium dyshomeostasis and synaptic morphological changes, we acutely exposed the healthy mouse brain, at 3 to 6 months of age, to naturally occurring soluble oligomers and investigated their effect on calcium levels using in vivo multiphoton imaging.

**Results:**

We observed a dramatic increase in the levels of neuronal resting calcium, which was dependent upon extracellular calcium influx and activation of NMDA receptors. Ryanodine receptors, previously implicated in AD models, did not appear to be primarily involved using this experimental setting. We used the high resolution cortical volumes acquired in-vivo to measure the effect on synaptic densities and observed that, while spine density remained stable within the first hour of oAβ exposure, a significant decrease in the number of dendritic spines was observed 24 h post treatment, despite restoration of intraneuronal calcium levels at this time point.

**Conclusions:**

These observations demonstrate a specific effect of oAβ on NMDA-mediated calcium influx, which triggers synaptic collapse in vivo. Moreover, this work leverages a method to quantitatively measure calcium concentration at the level of neuronal processes, cell bodies and single synaptic elements repeatedly and thus can be applicable to testing putative drugs and/or other intervention methodologies.

**Electronic supplementary material:**

The online version of this article (doi:10.1186/s13024-017-0169-9) contains supplementary material, which is available to authorized users.

## Background

Alzheimer’s disease (AD) is a devastating age-related neurological disorder characterized by the accumulation of extracellular amyloid β (Aβ) peptides and intraneuronal neurofibrillary tangles. Fibrillar amyloid plaques constitute a major diagnostic hallmark of the disease and correlate with deleterious local effects including gliosis, neuritic dystrophies and synapse loss [1, 2]. However, accumulating data suggest that small soluble aggregates of Aβ (oligomers, oAβ) mainly contribute to neuronal toxicity [3–7], accumulating in the vicinity of senile plaques and at the synapse [8]. Despite these important findings, no direct evidence has specifically linked the presence of oligomers in their natural form with neuronal dysfunction and spine morphological changes in vivo. While synthetic Aβ peptides have been extensively used in the past, they showed a relatively weak potency as compared with “native” oligomers from AD brains in physiological concentrations. In transgenic mice, the concomitant presence of full-length human APP and its downstream catalytic fragments has prevented us from specifically addressing oAβ-related neurotoxicity. We therefore developed an assay to directly examine the consequences of naturally occurring oAβ on the intact mouse brain using longitudinal multiphoton calcium imaging, and observed acute changes in calcium homeostasis, followed by synaptic loss.

The calcium hypothesis of AD argues that Aβ peptides impact neuronal calcium homeostasis, leading to synaptic failure, spine loss, neural network disruption and cognitive deficits [9–11]. Indirect evidence suggests that calcium alterations may be an early neuropathological change associated with mild cognitive impairment in AD patients [12–14]. Aβ-induced stress has been experimentally linked with abnormal activation of calcium dependent molecular cascades [5, 15], alteration of long term potentiation [16] and memory deficits [16, 17]. The development of new calcium sensors and their combination with in vivo two-photon imaging has further allowed a direct evaluation of calcium changes in AD mouse models, demonstrating in particular a dramatic increase in resting calcium levels in a subset of neurites surrounding amyloid deposits [18]. Increased proportions of hypoactive and hyperactive neurons have been reported in transgenic animals, the latter being found in clusters near amyloid plaques [19]. At the network level, a gradual decline in visual sensory performances has been observed in APP mice, a phenomenon correlated with the amyloid load [20–22]. Intriguingly, most of these studies did not detect changes before amyloid deposition, with only one previous work reporting hyperactivity associated with synthetically cross-linked dimers in vivo [6]. Taken together, testing the hypothesis that physiologically relevant oAβ causes local neuritic calcium elevation in the intact brain has not been achieved.

In the present study, we investigated the effect of naturally occurring soluble oAβ on neuronal calcium homeostasis in adult wild type animals. Acute application of oAβ, prepared from conditioned media isolated from transgenic primary neurons (TgCM), to the mouse brain surface dramatically increased neuronal resting calcium levels, whereas no change could be observed when conditioned media collected from the wild-type littermates cultures (WtCM) was used. This effect was abolished after immunodepletion of Aβ, thus specifically linking the presence of neurotoxic oAβ with intraneuronal calcium dysregulation. Because MK-801 efficiently blocked oAβ-induced Ca^2+^ increase, we also concluded that calcium dysregulation was primarily mediated through NMDA receptors, whereas antagonists of intracellular calcium channels were ineffective. In this regard, the current work addresses a long-standing controversy about whether oAβ activates the release of internal calcium stores or opens cell surface receptors. In addition, while the calcium rise was rapidly buffered, synapse loss was only detected after 24 h, demonstrating that oAβ-induced functional alterations precede morphological changes in vivo. Lastly, the approach used here to repeatedly measure calcium concentrations in vivo in the different neuronal compartments, including soma, dendrites and synaptic elements, may find broad applications to evaluate modulators of oAβ neurotoxicity and investigate the impact of various soluble neurotoxic species relevant in other neurodegenerative diseases.

## Methods

### Animals

Tg2576 males (Taconic Farms), which heterozygously overexpress human APPswe under the PrP promoter, were mated with wild-type females (B6;SJL) for preparation of primary cortical neurons. In vivo imaging experiments were performed using wild-type C57BL/6 males (3–6 months of age, Charles River). Each experimental group consisted of 3 to 6 animals.

### Preparation of wild type and transgenic neuronal conditioned media and Aβ immunodepletion

Cortical neurons were prepared as previously described [15] and plated on 35-mm culture dishes coated with poly-D-lysine (Sigma-Aldrich) and maintained in Neurobasal/B-27 media. Tissue from each embryo was collected and used for genotyping using PCR. Conditioned media was collected at 14 days in vitro (DIV) from either transgenic cultures (TgCM) or their wild-type littermates (WtCM). The concentrations of Aβ40 and Aβ42 were quantified by a mouse/human ELISA kit (Wako).

Immunodepletion of human Aβ from TgCM was performed overnight using the mAb 6E10 (Covance) and protein G sepharose beads (Sigma-Aldrich). Briefly, to avoid non-specific binding, protein G beads were washed with cold Neurobasal medium and incubated with 1 ml of TgCM for 30 min at 4 °C while shaking. The mixture was then centrifuged and the beads were discarded. The supernatant was further incubated with 100 μl pre-washed protein G beads and 9 μg of 6E10 antibody for 5 h at 4 °C while shaking. The supernatant was then collected and Aβ content was measured using a human/mouse ELISA kit (Wako).

### Size exclusion chromatography of condition media

Size exclusion chromatography (SEC) was performed as previously described [5]. Briefly, TgCM or WtCM was collected from 14 DIV neurons and centrifuged at 3,000 g at 4 °C in Amicon Ultra-15ML 3 K to concentrate proteins approximately fivefold. Concentrated TgCM or WtCM was separated by tandem Superdex 75 10/300 GL columns in 50 mM ammonium acetate, pH 8.5, with AKTA purifier 10 and dialyzed against PBS. Aβ40 content within the different fractions was measured after dialysis of the fractions with PBS using mouse/human ELISA kit (Wako). Fractions were also subjected to oligomer-specific ELISA kit (IBL). Assay was performed according to the manufacture instructions.

### Viral vector construction and production

The YC3.6 cDNA was cloned ﻿into an AAV2 backbone, under a hybrid cytomegalovirus (CMV) immediate-early enhancer/chicken β-actin promoter/exon1/intron and before the woodchuck hepatitis virus posttranscriptional regulatory element (WPRE). High titers of AAV serotype 8 were produced using the triple transfection protocol by both the Harvard and UPENN Vector Cores. Viruses were tittered by quantitative PCR and the final concentrations of these AAV viral stocks reached 4x10^12^ vg/ml.

### Stereotactic intraparenchymal injection of AAV-CBA-YC3.6

Stereotactic intracortical injections of AAV-CBA-YC3.6 were performed as described previously [23]. Briefly, animals were anesthetized by intraperitoneal injection of ketamine/xylazine (100 mg/kg and 50 mg/kg body weight, respectively) and positioned on a stereotactic frame (Kopf Instruments). Injections of vector were performed in the somatosensory cortex with 3 μl of viral suspension using a 33-gauge sharp needle attached to a 10-μl Hamilton syringe (Hamilton Medical), at a rate of 0.1 μl/min. Stereotactic coordinates of the injection sites were calculated from bregma (anteroposterior −1 mm, mediolateral ± 1 mm and dorsoventral − 1 mm).

### Cranial window implantation

One month after injection of AAV-CBA-YC3.6, mice were anesthetized with isoflurane (1–1.5%) and a cranial window was implanted by removing a piece of skull above the somatosensory cortex and replacing it with a 8 mm diameter cover glass (as described previously [24]). For all experiments, the dura mater was removed prior to window installation. Texas Red dextran (70 kDa; 12.5 mg/ml in PBS; Molecular Probes) was injected into a lateral tail vein before imaging to provide a fluorescent angiogram. Mice were imaged first to determine resting calcium at baseline. The window was then opened and closed again along with either WtCM, TgCM or depleted-TgCM diluted 1:1 with PBS (final volume applied was 50 μl). The window, containing the applied solution, was then resealed with dental cement. One hour later, each animal was reimaged at the same fields of view to determine the relative changes in [Ca^2+^] (ΔR/Ri) _i_. To test the effect of different calcium channel inhibitors, both dantrolene (5 μM) and MK-801 (5 μM) were incubated on the brain for 10 min before topical application with TgCM and each inhibitor mixed together for another hour.

### In vitro resting calcium imaging and measurements

To evaluate the effect of calcium channel antagonists in vitro using Tg2576 primary neurons, we used two different ratiometric calcium indicators, indo-1 AM (Life technologies) and YC3.6. For indo 1- experiments, 10–12 DIV cultures were incubated with 5 μM indo-1 AM for 45 min before imaging. For YC3.6 experiments, cells were transfected at 7 DIV with a pAAV-CBA-YC3.6 plasmid, using Lipofectamine 2000 (Life technologies), and imaged 2–3 days later. In both cases, hAPPsw transgenic neurons were imaged before and after bath application of an antagonist to calcium channels, using an LSM510 confocal microscope (Zeiss) with an environmental chamber that maintains constant conditions of 37 °C and 5% CO_2_. In the case of indo-1, a chameleon laser (Coherent, Inc) generated two photon excitation at 750 nm and emitted light was collected at 390–465 nm and 468–533 nm corresponding to the calcium bound and unbound dye, respectively. For YC3.6 experiments, single photon excitation was used at 458 nm using an argon laser, and emitted light was collected using the 435–485 BP and 505 LP filters for detection of CFP and YFP fluorescence, respectively. We tested the effect of the following agents on resting calcium levels in transgenic neurons: NiCl_2_ (100 μM), nifedipine (5 μM), ω-conotoxin (1 μM), MK-801 (5 μM), 2-APB (100 μM) and dantrolene (10 μM). All pharmacological agents were purchased from Sigma-Aldrich and dissolved either in double distilled water (ddw) or DMSO according to the manufacturer instructions. For indo-1 AM experiments, 5–6 fields of view were imaged randomly before and 10–15 min after bath application of the inhibitor. During culture experiments with YC3.6, a limited number of neurons were transfected, allowing us to reimage the same cells before and after treatment and thus to calculate the relative change in YFP/CFP ratio for each cell (ΔR/Ri). Each experiment was repeated over 3–4 independent trials.

### In vivo multiphoton imaging

For in vivo calcium imaging, an Olympus FluoView FV1000MPE multiphoton laser-scanning system mounted on an Olympus BX61WI microscope and an Olympus 25× dipping objective (NA = 1.05) were used. A DeepSee Mai Tai Ti:sapphire mode-locked laser (Mai Tai; Spectra-Physics) generated two-photon excitation at 860 nm, and detectors containing three photomultiplier tubes (Hamamatsu, Ichinocho) collected emitted light in the range of 460–500, 520–560 and 575–630 nm. Mice were placed on the microscope stage and heated using a heating pad with feedback regulation from a rectal temperature probe (Harvard apparatus). Four to six cortical volumes (Z-series, 127 μm × 127 μm, 2 μm slices, depth of 200–300 μm) were taken per mouse, before and after topical application of conditioned media, at a resolution of 512 × 512 pixels. Because of those imaging settings (small field of view and slow scan speed) we mostly did not record any type of neuronal oscillations (1.66 s per frame > 1Hz). CFP and YFP PMTs settings remained unchanged throughout the different imaging sessions. Laser power was adjusted as needed.

### Image processing and analysis

#### Cell cultures

Cells were imaged using a confocal Zeiss LSM510 system. Images were processed and analyzed using ImageJ software (https://imagej.nih.gov/ij/). For indo-1 experiments, single plane images of channels corresponding to calcium bound and unbound dye were obtained. For each channel, the background, corresponding to the mode of the image, was subtracted and a median filter with radius 2 was applied before dividing the emitted fluorescence intensity of the calcium bound channel by the unbound dye channel, thus creating a ratio-image. For YC3.6 experiments, single channels corresponding to CFP and YFP fluorescence were processed in the same way described for indo-1, creating a YFP/CFP ratio image. In both cases, cell bodies were manually selected using the free hand tool, before and after application of an antagonist. The selected regions of interest were then placed on the ratio image and measured. To convert the measured ratios, either for indo-1 or YC3.6, into calcium concentrations, calibration experiments were performed in CHO cells using each calcium indicator and solutions with either zero or 39 μM calcium in the presence of ionomycin (20 μM, Life Technologies). This allowed the determination of the Rmin and Rmax for each indicator (indo-1: Rmin = 0.54, Rmax = 3.04, YC3.6: Rmin = 0.19, Rmax = 1.618). Ratios were then converted to actual calcium concentration using the indo-1 K_D_ (250nM) and the K_D_ of YC3.6 (278nM) with standard ratiometric equations [25, 26]:

$$ \left[ Ca2+\right] i= K d\hbox{'}{\left(\frac{R- R min}{ R max- R}\right)}^{\raisebox{1ex}{$1$}\!\left/ \!\raisebox{-1ex}{$ n$}\right.}; K{d}^{\hbox{'}}= K d{\left(\frac{Sf2}{Sb2}\right)}^{\raisebox{1ex}{$1$}\!\left/ \!\raisebox{-1ex}{$ n$}\right.} $$

*Sf2* and *Sb2* represent the intensities at calcium free and calcium bound of the denominator (i.e. YFP or Indo-1 at 400 nm).

Pseudocolored images presented were created in Matlab based on each indicator ratio, which was then converted to calcium concentration using the empirical Rmin and Rmax and assigned to the jet colormap. We used the ratio values to supply the Hue and Saturation (color) and the reference image to supply the Value (intensity).

#### In vivo

Image stacks were processed in Image J as follows: the background of CFP and YFP channels, corresponding to the mode of the deepest slice of the z-stack, was subtracted, and a median filter (radius 2) was applied before dividing the emitted fluorescence intensity of YFP by CFP, thus creating a ratio image. Neurites, cell bodies and dendritic spines were identified and selected using the YFP images. The YFP/CFP ratio was then measured for each ROI using the ratio images. YFP/CFP ratios were converted to [Ca^2+^] _i_ with standard ratiometric equations (see above) using the in situ K_D_ and Hill coefficient of YC3.6 we have previously determined [18]. We established the dynamic range of the indicator in-vivo, using the same in vivo experimental settings as all our in vivo experiments. Rmin and Rmax were calculated using the lowest 2.5% of plaque-associated neuritic dystrophies (in AD transgenic mice) and the upper 2.5% of neurites in a dying mouse, respectively (Rmin = 0.79, Rmax = 4.5).

### Dendritic Spine analysis

2D projections of YFP-filled neurites were obtained in ImageJ. Dendrites of at least 20 μm long with prominent dendritic spine protrusions, were considered for analysis. Spines were selected manually and the relative changes in spine density were calculated for each neurite as compared with the average spine density before treatment for each animal. The nature of the treatment was kept blinded until statistical analyses.

### Statistical analyses

To assess whether ΔR/Ri values were different between the treatment groups in Neurite, Soma and Spines, a linear mixed effects model was fitted with treatment group as fixed effect and mouse as random effect. The same model was used for the analysis of Spine density.

To assess whether there was a difference between before vs. after CM application measurements in each group separately, a linear mixed effect model was fitted for each treatment group predicting the difference (after-before) with a fixed effect of an intercept only and a random effect of mouse.

YFP/CFP ratios were pooled and presented in histograms. Histograms for each group before treatment was fitted with a normal distribution curve using the Graph Pad curve fitting tool. Fisher exact test was used for comparison of calcium overload before and after treatment within each group. For the cell culture data, one way ANOVA with post hoc Dunn’s multiple comparison test was used for comparison of ΔR/Ri for each group and its vehicle treatment group (ddw or DMSO).

## Results

### Acute exposure of naturally occurring oAβ to the healthy living brain disrupts intraneuronal calcium homeostasis and synaptic integrity in vivo

Previous works have established that resting calcium levels and neuronal function are affected in transgenic mice with plaques, whereas no change was observed before amyloid deposition in most studies [18–21]. We have previously observed that TgCM treatment of wild type cortical neurons in vitro induced AD-like morphological changes that include calcium dyshomeostasis, calcineurin activation [15], dendritic spine loss and neuritic simplification [5].

To appreciate the specific contribution of soluble oAβ species to calcium dysregulation, we designed an acute in vivo assay of Aβ neurotoxicity by topically applying oAβ-enriched medium on the cortical surface of wild-type C57BL/6 mice. Culture medium from primary cortical neurons derived from Tg2576 embryos was used as a source of soluble oAβ (termed TgCM), which mainly contains low molecular weight oligomers (dimers to tetramers) as evidence from size exclusion chromatography (SEC) followed by Aβ40 ELISA (Additional file 1: Figure S1) [5, 15, 27]. To validate the presence of oAβ in the TgCM, the different SEC fractions were also subjected to oligomeric Aβ ELISA (IBL) that utilizes the same antibody for capture and detection. Additional file 1: Figure S1B shows the presence of oAβ (at much lower levels compared to total Aβ40 signal) in both the high and low molecular weight fractions. Higher levels were detected between fractions 11 and 16, which corresponds to 13.7 kDa and smaller, very low levels were detected at fractions 25–28, which correspond to 6.5 kDa and lower. However, the signal at these fractions was very close to the limit of the ELISA kit sensitivity. This data suggest that that most Aβ oligomers within the TgCM are of low molecular weight.

Conditioned media from non-Tg neurons (termed WtCM) and Aβ immunodepleted TgCM were used as controls. Within different batches of TgCM, Aβ40 and Aβ42 concentration respectively reached 4-8nM and 130–260pM, which corresponds to the levels detected in human cerebral spinal fluid [28]. Additionally, the concentration of oAβ in the TgCM reached 400pM to 600pM, representing 7.5–10% of the total amount of Aβ_40_. The content of murine Aβ40 in several batches of WtCM ranged from 200pM to 500pM while murine Aβ42 could not be detected.

**Fig. 1:**
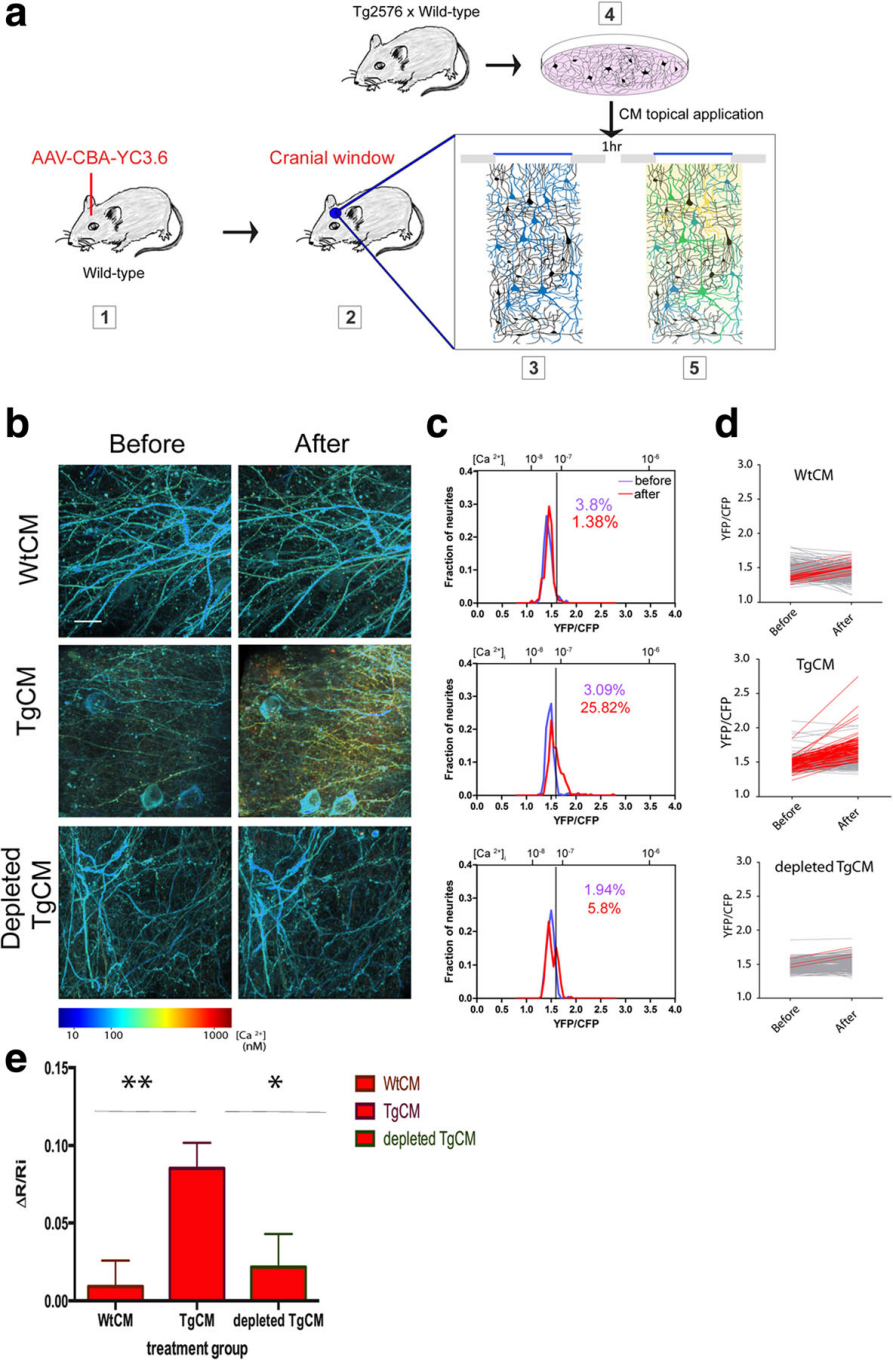
Oligomeric Aβ induces calcium overload in the healthy mouse brain. **a** A new experimental setting was designed to specifically address the effect of oAβ on calcium homeostasis in vivo. Wild-type C57BL/6 mice were injected with an AAV-CBA-YC3.6 and a cranial window was installed 4 weeks later. After a first imaging session at baseline, the window was opened and WtCM, TgCM or depleted TgCM was topically applied for 1 hour before reimaging again the exact same fields of view. **b** Neurites, consisting of dendrites and axons, filled with YC3.6 were imaged before and after treatment with TgCM, WtCM or depleted-TgCM. Images were pseudocolored according to calcium concentration (Scale bar = 20 μm). **c** Individual neurites were selected for measuring YFP and CFP ratio and calculating resting calcium levels. Distribution curves of [Ca^2+^] _i_ (YFP/CFP ratio on the lower x-axis and [Ca^2+^] _i_ on the upper x-axis) are presented before (*purple curve*) and after treatment (*red curve*) for each experimental group. Acute exposure to TgCM, but not to WtCM or immunodepleted-TgCM, resulted in a shift in the [Ca^2+^] _i_ distribution towards higher ratio values, i.e. higher calcium concentrations. The *black* line indicates the threshold for calcium overload which represents 2 SD above the mean of ratios measured from all mice before treatment. The percentage of neurites with calcium overload before (*purple*) and after (*red*) treatment is noted on the graphs. Calcium overload was only significantly increased in the TgCM treated group from 3.09 to 25.82% (*p* < 0.0001 Fisher exact test). **d** YFP/CFP ratios before and after treatment with WtCM, TgCM or depleted TgCM represented for each neurite. The *red* traces account for the neurites that showed an increase of ≥10% in YFP/CFP ratio after topical application of CM. **e** The relative change in ratio was calculated for each neurite and the mean + SEM of each group is presented (linear mixed effects model fitted with treatment group as fixed effect and mouse as random effect ** *p* = 0.0012, *p = 0.0183)

**Fig. 2:**
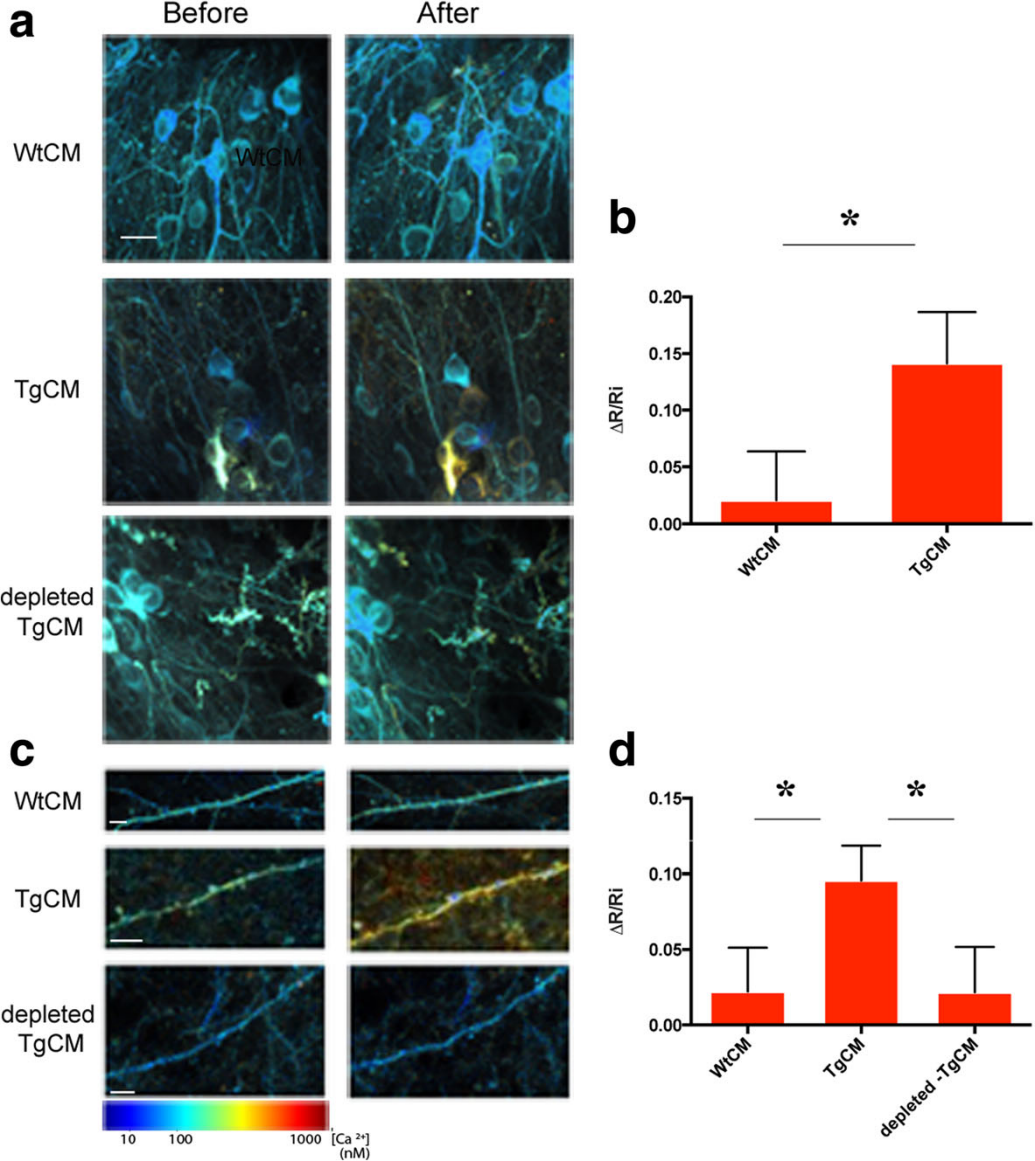
Oligomeric Aβ induces calcium overload in neuronal cell bodies and dendritic spines. Neuronal cell bodies (**a**,**b**) or dendritic spines (**c**,**d**) were imaged before and 1 h after treatment with conditioned media, and the YFP/CFP ratio and resting calcium were calculated for each compartment. Images were pseudocolored according to calcium concentrations. **b** In cell bodies, TgCM, but not WtCM, induced an increase in resting calcium. (TgCM: *n* = 165 cells, WtCM: *n* = 157 cells, in 5 mice, pair-wise comparison of the relative change in YC ratio, * *p* = 0.0458). **d** TgCM effect on YC ratio in dendritic spines followed the same trend as neurites and was significantly different from that of WtCM and depleted-TgCM (pair-wise analysis TgCM vs. WtCM * *p* = 0.0466; TgCM vs. depleted-TgCM * *p* = 0.0494; WtCM vs. depleted-TgCM *p* = 0.9916). Scale bars = 20 μm in (**a**) and 5 μm in (**c**)

To quantify absolute calcium levels in the brain, mice were injected with an AAV8 encoding the ratiometric calcium indicator yellow-cameleon YC3.6 [29]. Four weeks later, a craniotomy was performed above the somatosensory cortex and CFP and YFP fluorescent signals were measured. After evaluating the baseline neuronal resting calcium, the cranial window was reopened and either TgCM or WtCM, diluted 1:1 in PBS (final concentration of Aβ40 of 2nM in TgCM and 180pM in WtCM), was directly applied to the brain surface. Using the labeled brain vasculature as a guide, we were able to reimage the same field of view and calculate the relative changes in intracellular calcium. We imaged the first 300 μm of the cortical surface, which consist of neurites and cell bodies located in layers II/III (Figs. 1a and 2a).

Using the YC3.6 K_D_ (278nM) previously calculated in cultures [18] and the dynamic range of the indicator (Rmin and Rmax) obtained from the in-vivo data acquired using the same microscope under the same settings (see Methods), we converted YFP/CFP ratios into absolute calcium concentrations. Our measurements indicate that [Ca^2+^] i is tightly regulated in wild type mice and reaches 70 ± 4nM on average. The values of [Ca^2+^] i in neurites in wild type brain generally follow a normal distribution (Fig. 1c, r^2^ = 0.99). As expected, only rare neurites would be classified as having calcium overload at baseline (threshold defined as [Ca^2+^] i greater than two SD above the mean of all neurites in the first imaging session, 90nM). Topical application of TgCM to the healthy brain progressively increased the YFP/CFP ratios and thus [Ca^2+^] i, which reached a peak after an hour before returning to baseline levels by 5 h (Additional file 2: Figure S2). One-hour exposure with TgCM induced a dramatic elevation of intraneuronal calcium, as evident in the pseudocolored images and the right shift of the calcium distribution curves towards higher YFP/CFP ratio values (Fig. 1b, c). TgCM incubation resulted in a greater number of calcium-overloaded neurites (threshold for calcium overload is represented by a black line; *n* = 295 neurites in 5 mice, calcium overload: before = 3.09%, after = 25.82%, *p* < 0.0001 Fisher exact test). Conversely, when mice were incubated with WtCM, no significant change in calcium levels was observed (*n* = 409 neurites in 5 mice, calcium overload: before = 3.8%, after = 1.38%, *p* = 0.643 Fisher exact test). To demonstrate that TgCM-induced elevation of [Ca^2+^] _I_ was directly related to the presence of Aβ peptides in the medium, we immunodepleted human Aβ from the TgCM and followed the same experimental settings. Immunodepletion of Aβ with 6E10 antibody effectively removed about 80% of Aβ present in TgCM (initial concentration of Aβ40 in TgCM: 4.1nM, after immunodepletion: 0.8nM). The effect of TgCM was abolished after immunodepletion and only a modest non-significant trend of calcium overload was detected (*n* = 206 neurites in 3 mice, calcium overload: before = 1.9%, after = 5.8%, *p* = 0.07 Fisher exact test, comparison of before and after YFP/CFP ratios *p* < 0.2847).

Comparing resting calcium before and after treatment within the same neuronal processes, we observed that the YFP/CFP ratios were increased by 10% or more for the vast majority of TgCM-treated neurites, whereas such a change was only occasionally observed after WtCM and depleted-TgCM incubation (Fig. 1d). From these values we calculated the relative change in YFP/CFP ratio for each neurite (ΔR/Ri, Fig. 1e) and showed that TgCM induced an average increase in the YFP/CFP ratio of 8.5 ± 1.6% (from 70nM to about 85nM). Application of TgCM was significantly different from that of WtCM and depleted-TgCM while the later two groups were not different from each other (Fig. 1e, linear mixed effect model. Overall change between the groups *p* = 0.0029, post hoc comparisons between every 2 groups: *p* = 0.0012 TgCM vs. WtCM; *p* = 0.0183 TgCM vs. depleted TgCM; *p* = 0.6507 WtCM vs. depleted-TgCM. *n* = 295 neurites within 5 mice for TgCM, 405 neurites within 5 mice for WtCM and 206 neurites picked from 3 mice for depleted-TgCM).

When only the overloaded neurites after TgCM treatment were analyzed, the average reached a mean change of 16.9 ± 4.8% (from 70nM to 100nM). Reimaging the same fields and analyzing the YFP/CFP ratios in the same neurites 5 h post treatment did not show any change from baseline (Additional file 2: Figure S2), suggesting that elevation in [Ca^2+^] _i_ was a transient event rapidly buffered in the healthy brain of wild type mice.

When cell bodies were selected (Fig. 2a and b), resting calcium concentration at baseline was lower than the level measured in neurites, averaging at 58nM. Exposure with oligomeric Aβ also disrupted calcium homeostasis in the neuronal soma as compared with WtCM treatment (ΔR/Ri : TgCM 14.4 ± 4.25%, WtCM 2.09 ± 4.2%, pair-wise comparison, *p* = 0.0458). However, we could not analyze the depleted –TgCM as we could trace cell bodies only in one of 3 mice tested. TgCM treatment effect was significantly different from that of WtCM.

**Fig. 3:**
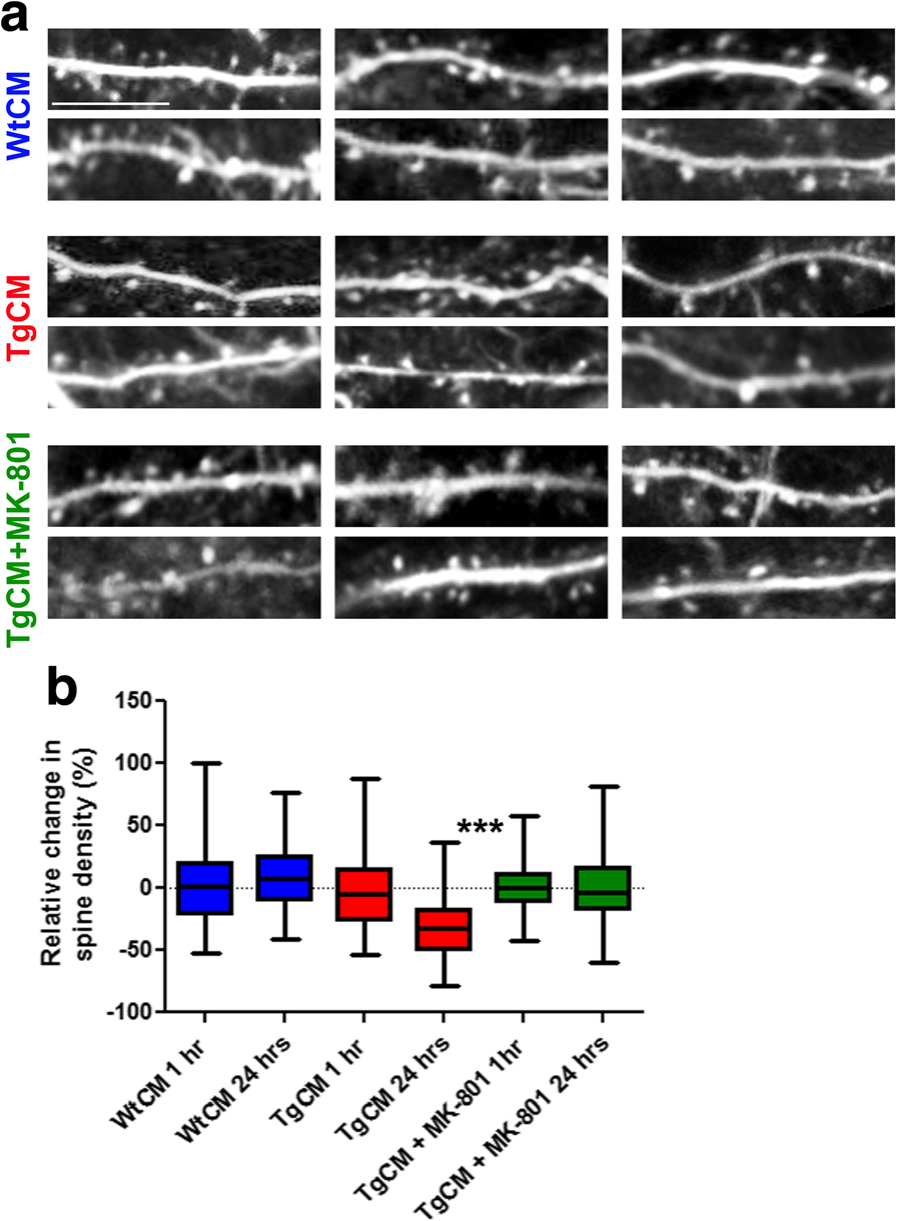
Oligomeric Aβ decreases spine density at 24 h. **a** Representative images of YFP-filled neurites and dendritic spines before and after 1 h or 24 h treatment with WtCM, TgCM and TgCM + MK-801 (Scale bar = 10 μm). **b** Box plots showing the relative changes in percentage of spine densities after treatment accordingly to the mean spine density calculated for each animal before treatment. Whiskers represent the minimal and maximal values of each experimental group (*n* = 3–4 mice per group. For WtCM: *n* = 58 neurites and 578 spines evaluated after 1 h and *n* = 91 neurites and 1382 spines evaluated after 24 h. For TgCM: *n* = 68 neurites and 760 spines evaluated after 1 h and *n* = 69 neurites and 635 spines evaluated after 24 h. For TgCM + Mk-801: *n* = 152 neurites and 854 spines evaluated after 1 h and *n* = 174 neurites and 1044 spines evaluated after 24 h. Linear mixed effects model was fitted with treatment group and time of imaging (as outlined in the graph) as fixed effect and mouse as random effect. (pair-wise comparisons: TgCM 1 h vs. 24 h: *** *p* < 0.0001, Wtcm 1 h vs. 24 h *p* = 0.3897, TgCM + MK-801 1 h vs. 24 h *p* = 0.6667)

Dendritic spines showed indistinguishable [Ca^2+^] i from that of the neurites (Fig. 2c, d; YFP/CFP ratios corresponding to about 72 ± 10nM and 70 ± 4nM in spines and neurites, respectively). Similarly to our observations in neurites, TgCM application led to a 9.6% ± 2.3% increase in calcium levels (comparison before and after TgCM: *p* = 0.0268). WtCM and depleted-TgCM application did not have any effect (before vs. after comparison; WtCM: *p* = 0.3736, depleted-TgCM: *p* = 0.5552). The effect of TgCM on the relative change in calcium was significantly different from that of WtCM and depleted TgCM, which did not differ from each others (pair-wise comparisons; TgCM vs. WtCM *p* = 0.0466, TgCM vs. depleted TgCM *p* = 0.0494, WtCM vs. depleted TgCM *p* = 0.9916). In contrast with the acute and dramatic impact of oAβ on intraneuronal calcium, no change in spine density was detected 1 h after treatment with TgCM (relative change compared with the average spine density before treatment: −0.2% ± 4.1%, *p* = 0.9502, 760 spines counted in 4 mice) or with WtCM (relative change compared with the average spine density before treatment: 2.2% ± 5.3%, *p* = 0.708 578 spines counted within 3 mice, Fig. 3). However, a robust decrease in the density of dendritic spines was observed 24 h after TgCM application (relative change compared with the average spine density before treatment: −28.7% ± 4.3%, *p* < 0.0001, *n* = 635 spines within 3 mice, Fig. 3), at a time when the calcium levels were restored to baseline (Additional file 2: Figure S2). By contrast, non-significant change in spine density was observed 24 h post application of WtCM (relative change compared with the average spine density before treatment (8.9% ± 4%, *p* = 0.0555, 1382 spines counted within 3 mice, Fig. 3), suggesting that this observation did not derive from the surgical procedure itself. Pair-wise analysis between the two groups (TgCM/WtCM) showed no difference at 1 h (*p* = 0.7463) and significant change in spine density at 24 h (*p* < 0.0001) after treatment.

### NMDA receptors mediate oAβ-induced increase in resting calcium

Intracellular changes in neuronal [Ca^2+^] _i_ can result from increased entry of extracellular calcium, leakage of intracellular calcium stores or loss of buffering capacity. To determine the molecular mechanisms underlying TgCM-induced calcium dysregulation, we examined these possibilities in vitro and in vivo.

**Fig. 4:**
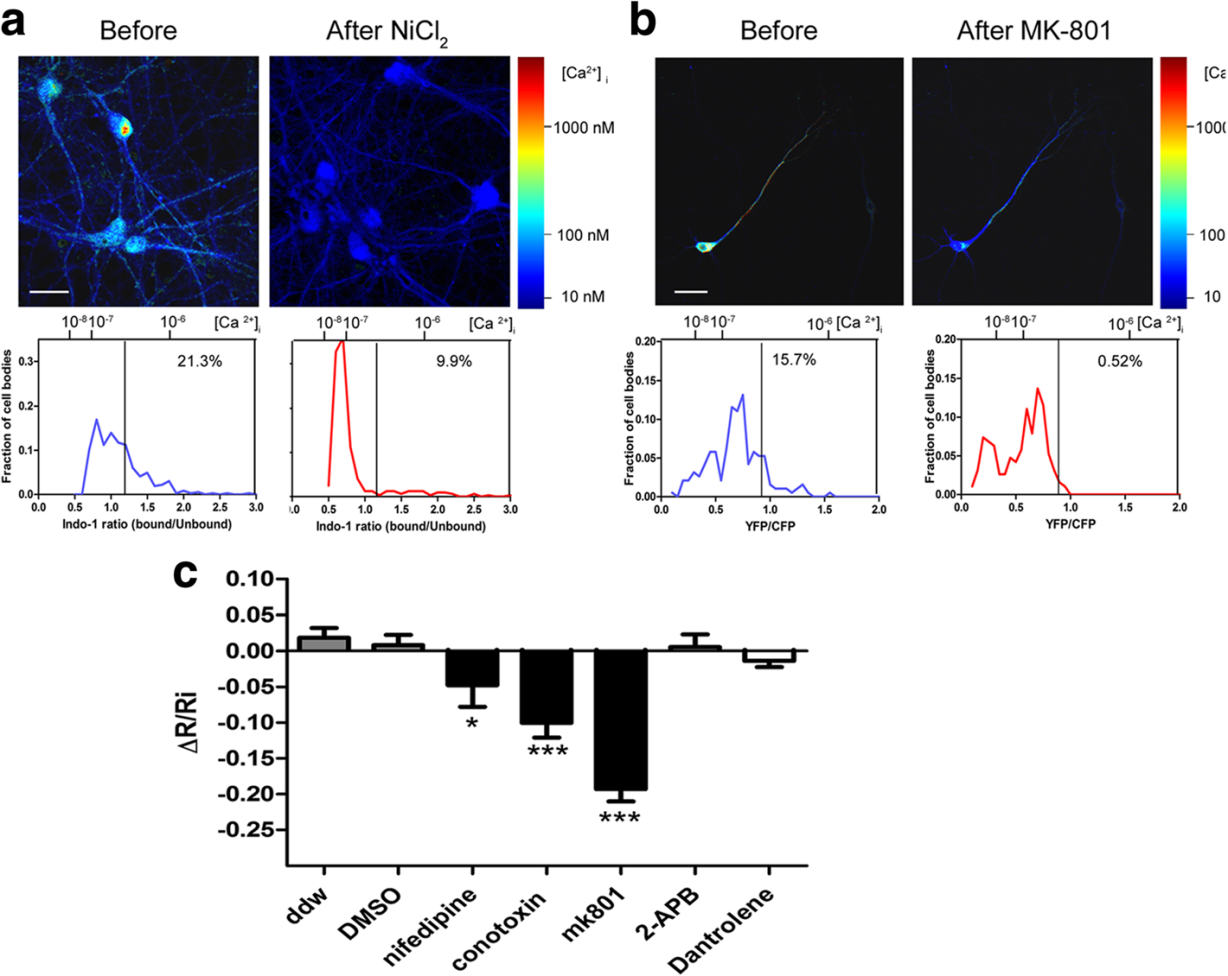
Extracellular calcium is the source of calcium overload in primary neurons expressing human APP. **a** Tg2576 neurons were incubated with the ratiometric calcium indicator indo-1 AM and then imaged before and after application of NiCl_2_ to block all voltage gate calcium channels. The ratios of bound vs. unbound indo-1 were calculated, converted to calcium concentration and pseudocolored using the jet color map. Distribution curves of [Ca^2+^] _i_ (bound/unbound ratio on the lower x-axis and [Ca^2+^] _i_ on the upper x-axis) are presented in the lower panel showing a wider distribution at baseline, which became more narrow and was shifted to the left after application of NiCl_2_. The *black* line indicates the threshold for calcium overload. The percentage of neurons with calcium overload, noted on the graphs before and after treatment, is significantly reduced by NiCl_2_ from 21.3 to 9.9% (*n* = 366 neurons before treatment and 321 after treatment in three separate experiments, Fisher exact test, *p* < 0.0001). **b** Neurons were transfected with YC3.6 and imaged before and after application of the NMDA receptor antagonist, MK-801. Ratios were converted to calcium levels and pseudocolored as in A. Upper panel shows resting calcium measured in the same neuron before and after inhibition of NMDA receptors. Distribution curves of [Ca^2+^] _i_ (YFP/CFP on the lower x-axis and [Ca^2+^] _i_ on the upper x-axis) are shown on the lower panel. Again, the wide distribution of calcium initially observed in transgenic neurons was shifted to the left after treatment with MK-801, dramatically reducing calcium overload from 15.7 to 0.5% (*n* = 189 neurons in 4 separate experiments, Fisher exact test *p* < 0.0001). **c** Summary bar graph for experiments with antagonists for different calcium channel measured with YC3.6 as described in B. The relative change in ratio (ΔR/Ri) after exposure with each inhibitor was calculated for individual cells and presented here as the group mean + SEM (each inhibitor was tested in three independent experiments: vehicles: ddw – *n* = 243, DMSO – *n* = 161; inhibitors: Nifedipine (L-type voltage channels) – *n* = 158, ω-conotoxin (N-type channels) – *n* = 141, 2-APB (IP_3_R) – 173, dantrolene (RyR) – 208). The effect of the treatment was tested by comparing each inhibitor to its appropriate vehicle using one way ANOVA with post hoc Dunn’s Multiple comparison test (* *p* < 0.05, ** *p* < 0.01, ***p < 0.0001). (Scale bar = 50 μm)

As a preliminary approach, we used cortical primary neurons cultured from Tg2576 embryos to perform an initial screen of various calcium channel inhibitors. These neurons chronically secrete human Aβ that oligomerize to form predominantly species from dimers to tetramers after 14DIV (Additional file 1: Figure S1). We have previously demonstrated that, similarly to wild type neurons treated with TgCM, Tg2576 neurons also exhibit spine loss and decreased neurite arborization [5, 15]. We assessed the calcium levels with two different calcium probes: YC3.6 and the small molecule ratiometric indicator indo-1/AM. To explore the contribution of the extracellular calcium pool and the role of voltage gated calcium channels (VGCC), we incubated the cells with indo-1 AM and imaged before and 10 min after exposure to 0.5 mM NiCl_2_. Transgenic neurons in culture presented a wide distribution of calcium concentrations (Fig. 4a, note the right tail of the distribution curve). Before treatment, 21.3% of transgenic neurons showed calcium overload (marked by a black line, R = 1.32, 467nM), similar to our previous in vivo observations using this APP mouse model [18]. A 10-min incubation with NiCl_2_ significantly decreased the resting calcium levels and reduced the percentage of calcium overloaded cells from 21.3% to 9.9% (Tg before: *n* = 366 neurons in 3 experiments. Tg after NiCl_2_: *n* = 321 neurons in 3 experiments, *p* < 0.0001, Fisher exact test). We then performed similar experiments with specific pharmacological blockers of several calcium channels (nifedipine for L-type VGCC, ω-conotoxin for N-type VGCC and MK-801 for NMDA-R), as well as inhibitors of intracellular calcium release from the ER (2-APB for IP_3_ receptors and dantrolene for ryanodine receptors). These experiments were performed in transgenic neurons transfected with YC3.6 and the sparse expression of YC3.6 allowed us to re-visit each cell after application and thus appreciate the relative change in ratio for each neuron (ΔR/Ri). As evident in Fig. 4c, vehicles alone (water or DMSO) did not affect the resting calcium in cells. We observed that N-type, L-type VGCC and NMDA receptor antagonist were effective in reducing [Ca^2+^] _i_ in the presence of oAβ by 4.7%, 10.05% and 19.31%, respectively (One way ANOVA with post hoc Dunn’s multiple comparison test for comparison across all groups: Nifedipine vs. DMSO: *p* < 0.05, ω-conotoxin vs. ddw: *p* < 0.0001, MK-801 vs. ddw: *p* < 0.0001). MK-801 led to the most dramatic effect (Fig. 4*b*, *c*), shifting the distribution curve to the left and reducing calcium overload from 15.7 to 0.52% (*p* < 0.0001 Fisher exact test). Dantrolene and 2-APB, ryanodine and IP_3_ receptors antagonists that block calcium release from the ER, did not alter the resting calcium levels of Tg primary neurons. The moderate decrease in [Ca^2+^] _i_ observed when nifedipine or ω-conotoxin along with the more dramatic effect of MK-801 strongly support a major role for extracellular calcium as the source for calcium overload.

**Fig. 5:**
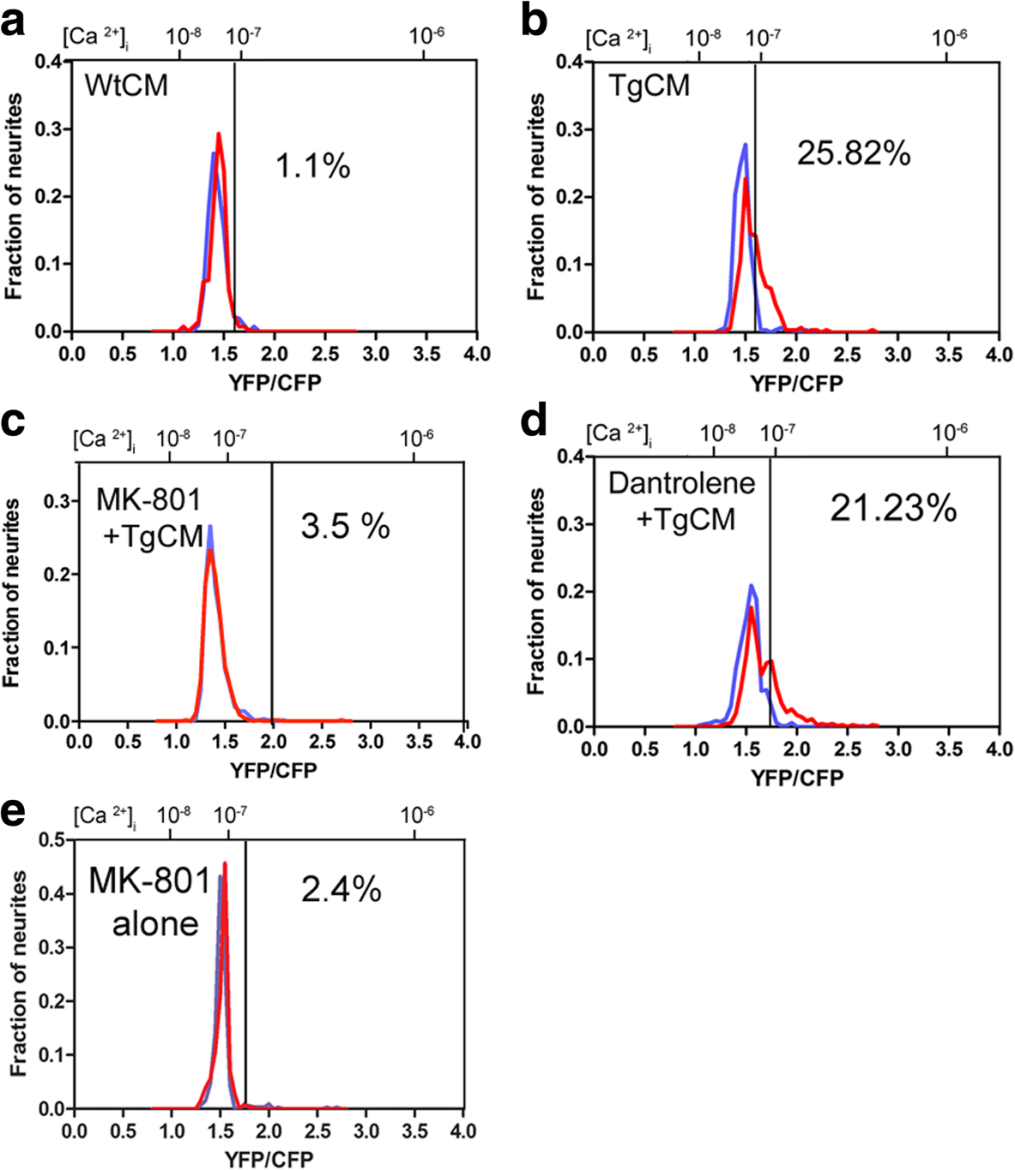
Oligomeric Aβ induces calcium overload through activation of NMDA receptors in vivo. The antagonists MK-801 and Dantrolene were applied 10-min before and during TgCM application. Distribution curves of [Ca^2+^] _i_ (YFP/CFP ratio on the lower x-axis and [Ca^2+^] _i_ on the upper x-axis) are presented before (*purple curve*) and after treatment (*red curve*) for mice treated with WtCM (**a**) TgCM (**b**) MK-801 + TgCM (**c**), dantrolene + TgCM (**d**) and MK-801 alone (**e**). The *black* line indicates the threshold for calcium overload. The percent of overloaded neurites is noted on each graph. Treatment with MK-801, but not of dantrolene, abolished the effect of TgCM on resting calcium (pair-wise comparisons of the relative change in YC ratio: TgCM vs. TgCM + MK-801 *p* = 0.0221; TgCM vs. TgCM + Dantrolene *p* = 0.7656; TgCM + MK-801 vs. TgCM + Dantrolene *p* = 0.0077)

To test if oAβ-induced calcium dysregulation also relies on extracellular stores and NMDAR activation in vivo, MK-801 (5 μM) was applied on the surface of the brain for 10 min before and along with the application of TgCM. As shown in Fig. 5, TgCM failed to affect resting calcium in mice pretreated with MK-801 (ΔR/Ri comparing before and after MK-801 + TgCM treatment: *p* = 0.7719). The percentage of calcium overloaded neurites also remained unchanged after MK-801/TgCM application (1.67 to 3.5%, *n* = 656 neurites from 7 mice, *p* = 0.0548 Fisher exact test), while TgCM alone resulted in 25.8% of neurites showing calcium overload (*p* < 0.0001, Fisher exact test). Additionally, we did not see any significant spine loss 1 h or 24 h after topical application of MK-801/TgCM (relative change compared with the average spine density before treatment: at 1 h: 0.4% ± 3.3%, *p* = 0.9123, and at 24 h: 2.4% ± 3.3; *n* = 4 mice), implying that the presence of MK-801 also counteracted the deleterious impact of oligomeric Aβ species on synapse loss previously observed with TgCM alone at 24 h. Importantly, MK-801 alone did not significantly changed calcium overload (2.79% to 2.4% overload before and after, *p* = 0.37, fisher exact test, *n* = 322 neurites in 3 mice Fig. 5e), thus demonstrating that the impact of this inhibitor was directly associated with oAβ-dependent activation of NMDAR in the first place. In contrast, treatment with dantrolene (5 μM) did not counteract the effect of TgCM (ΔR/Ri comparing before and after dntrolene + TgCM treatment: *p* = 0.0003).As evident by the change in the shape of the neurite distribution curve and the increased percentage of calcium overloaded neurites (from 2.07 to 21.23%; *n* = 674 neurites from 4 mice, *p* < 0.0001 Fisher exact). To conclude, MK-801 and dantrolene led to an opposite effect on resting calcium once applied to the brain along with TgCM (pair-wise comparison TgCM + MK-801 vs. TgCM + dantrolene: *p* = 0.0077), suggesting that extracellular soluble amyloid species initially act through activation of these calcium channels in vivo, while the calcium retained in the ER does not seem to play a primary role.

## Discussion

Disruption of intracellular calcium balance has been invoked as a potential mechanism of physiological and pathophysiological changes associated with normal aging [30] and, to a greater extent, with a variety of neurologic disorders including schizophrenia [31], bipolar disorder [32], autism spectrum disorder [33], Parkinson’s disease [34, 35], Huntington disease [36] and AD [10, 11, 37–39]. The relationship between the pathological hallmarks of AD and abnormal calcium homeostasis has been a focus of investigation, and while neurofibrillary tangles seemed to affect neither resting [Ca^2+^] _i_ [40] nor neuronal responses to stimuli [41], the amyloid pathology has been linked to increased resting calcium [18], neuronal hyperactivity [19] and a gradual decline in neuronal performance in response to visual stimuli [20, 21].

Accumulating evidence suggests that soluble oAβ are more potent neurotoxic species than Aβ fibrils, leading to synaptic and neural network dysfunction [3, 4, 42–44]. Though the effect of different species of oAβ has been tested in culture models, studying their relevance in vivo has been challenging because of the concomitant presence of amyloid plaques [8, 45]. In this study, we specifically investigated the effect of naturally secreted soluble species of Aβ, primarily oAβ, on calcium homeostasis in the healthy living brain. We utilized conditioned media collected from APP transgenic cortical neurons as the source of oligomeric Aβ. Unlike synthetic Aβ, which generates oligomers and fibrils within minutes to hours, naturally secreted Aβ peptides mainly assemble into low molecular weight species from dimers to tetramers. We demonstrated that, when oAβ was acutely applied to the cortical surface of the healthy mouse brain after cranial window implantation, the resting calcium levels were dramatically increased in all cellular compartments including neuronal soma, processes and dendritic spines. While it is conceivable that the surgical procedure itself sensitizes neurons towards Aβ neurotoxicity, the calcium rise could not be attributed solely to the experimental approach itself. Indeed, removal of Aβ from the conditioned media or, alternatively, acute application of conditioned media from wild type neurons, abolished this effect. Aβ-induced elevation of resting calcium was significant 1 h after application but could no longer be detected in the same brains after 5 h, suggesting clearance of the peptide assemblies, and/or recovery via intracellular buffering mechanisms. Interestingly, the relatively fast increase of intraneuronal calcium levels after exposure to Aβ leads to long-lasting morphological alterations with a significantly decreased spine density detected only 24 h after treatment with TgCM while no change could be detected at 1 h. The extent of oAβ-induced spine loss is similar to what has been reported in the immediate vicinity of amyloid plaques, where high concentrations of oAβ are thought to exist [8, 45]. The difference in kinetics between oAβ-dependent calcium overload and synaptic collapse strongly argues in favor of a primary neuronal dysfunction that precedes morphological changes at the synapse, perhaps via activation of calcineurin or other calcium dependent signaling pathways [5, 15, 27]. This is of particular interest considering that spine loss is the parameter that correlates best with cognitive impairment in AD patients [46, 47].

Understanding the source of the calcium entry in the presence of oAβ and the channels involved is important as calcium influx through different channels may trigger various signaling cascades which independently impact gene transcription and neuronal homeostasis [48]. The role of extracellular calcium and its influx through the activation of NMDA receptors was demonstrated in vivo, as oAβ-dependent calcium rise was prevented by incubation with MK-801. Indeed, Aβ has been shown to reduce long term potentiation (LTP) and facilitate long term depression (LTD), processes that involved trafficking of glutamate receptors [49, 50]. In addition, prefibrillar amyloid and Aβ oligomers have been shown to directly interact with glutamatergic receptors, activate NMDAR and result in calcium increase in primary neurons [51–53]. While those in vitro studies have established a direct association between oAβ and NMDAR, our results do not exclude the possibility that intermediate steps also participate in the rise of intraneuronal calcium levels. For example, pathologically elevated levels of Aβ have been shown to block glutamate uptake at the synaptic cleft, leading to increased glutamate levels, which could also activate synaptic NMDA receptors [54]. Whether or not the activation of NMDAR is a direct or indirect effect of extracellular oAβ, our data strongly suggest that the rise in intracellular calcium via NMDAR may impair synaptic plasticity and spine density, and thus represent the initial steps in the pathological changes that occur in AD.

ER calcium stores have been suggested to play a role in regulation of Aβ production [55, 56] and calcium release from the ER via ryanodine and IP_3_ receptors has also been implicated in calcium dyshomeostasis in AD [11, 57, 58]. Specifically, chronic treatment with the ryanodine receptor antagonist, dantrolene, was shown to normalize ER calcium responses, improve cognitive deficits and modulate the amyloid pathology in mouse models of AD [59]. Conversely, our data suggest that oAβ initially facilitates calcium entry from extracellular pools, at least largely via NMDA receptors, whereas ryanodine receptors do not appear to play a role primarily. It is possible that the ER pools are involved in a second step in calcium-dependent calcium release from the ER, resulting in further rise in cytosolic calcium levels. In the brains of AD mouse models chronically exposed to Aβ oligomers and fibrils, the sustained increase in resting calcium [18] may result from a concomitant dysregulation of extracellular calcium entry or intracellular calcium buffering systems that further induce downstream events such as synapse loss and alterations in APP processing. This may not be the case in the healthy brain in which calcium is highly regulated, explaining why the effect of oAβ on [Ca^2+^] _i_ is not abolished by blocking RyR, as the initial rise in calcium is not dependent on ER stores.

Finally, we cannot exclude that exposure of the cortical surface to oAβ impacts intraneuronal calcium homeostasis via non-cell autonomous mechanisms, eventually involving the participation of astroglial cells as well [60]. The effect of oAβ on cultured astrocytes has indeed been investigated over the past [61, 62], showing in particular that oligomerized Aβ can induce astrocytic glutamate release in an α7nAChR-dependent manner, activate extrasynaptic NMDAR and lead to synaptic loss. In vivo studies in AD mice have revealed that calcium transients in astrocytes were more frequent and synchronously coordinated in transgenic animals as compared with controls [63, 64], an effect potentially mediated by paracrine purinergic signaling that could be blocked by P2Y receptors inhibitors [65]. However, most of the work conducted in animals has been done using transgenic mouse models, so the sole effect of oAβ on astrocytic calcium homeostasis remains to be elucidated. Additionally, studying the respective impact of oligomeric amyloid species concomitantly on neurons and astrocytes will give further information about the initial molecular mechanisms in play.

In light of these observations, it is clear that therapeutic strategies aimed at reducing or inhibiting calcium influx into cells through NMDAR may be of importance. In parallel, efforts to prevent or reduce the levels of soluble oAβ levels will likely lead to treatments that effectively delay or restore neuronal dysfunction in AD.

## Conclusions

The present study demonstrates that low-molecular weight species of Amyloid β can acutely disrupt neuronal calcium homeostasis in non-transgenic animals through the activation of NMDA receptors. Interestingly, although calcium levels are rapidly restored to baseline, decreased spine density is detected 24 h after exposure to oAβ, suggesting that calcium dyshomeostasis precedes synaptic morphological changes. This work implicates small oAβ species in the deleterious effects that occur during degenerative processes at the synapse.

## Additional files

Additional file 1: Figure S1. Biochemical characterization of Aβ oligomers present in Tg conditioned media (TgCM). **a** Representative profile showing the amount of Aβ40 detected by ELISA in each fraction after separation of the different oligomeric species present in TgCM (red bars) by size-exclusion chromatography (SEC, Superdex 75 SEC columns). The molecular weight markers (kDa) that ran at the same conditions are indicated above (arrowheads). **b** Measurements of oligomeric Aβ within each SEC fraction show that most of oAβ is of low molecular weight (fractions 12–18).. A smaller peak of high-molecular weight species was also detected in TgCM (fractions 2–6 in both ELISAs). (TIF 1492 kb)

Additional file 2: Figure S2. Time course of oAβ-induced increase in resting calcium levels after acute exposure with TgCM on a healthy brain. C57BL/6 mice were injected with AAV-CBA-YC3.6 into the somatosensory cortex and acutely exposed to TgCM containing oAβ. The changes in YFP/CFP ratios in the neurites were measured at baseline before treatment and 30 min, 1 h and 5 h after topical application of TgCM. A progressive increase in the levels of resting calcium (YFP/CFP ratio and the actual calcium levels on the lower and upper x-axis, respectively) was detected that reached a peak after 1 h (20.6% of overloaded neurites), before returning to baseline levels after 5 h (1.7% overloaded neurites). (TIF 37844 kb)
