# Supplementary figures and images for: Soluble oligomeric amyloid-β induces calcium dyshomeostasis that precedes synapse loss in the living mouse brain
Source: Mol Neurodegener. 2017 Mar 21;12:27. doi: 10.1186/s13024-017-0169-9 (PMC5361864; doi:10.1186/s13024-017-0169-9)

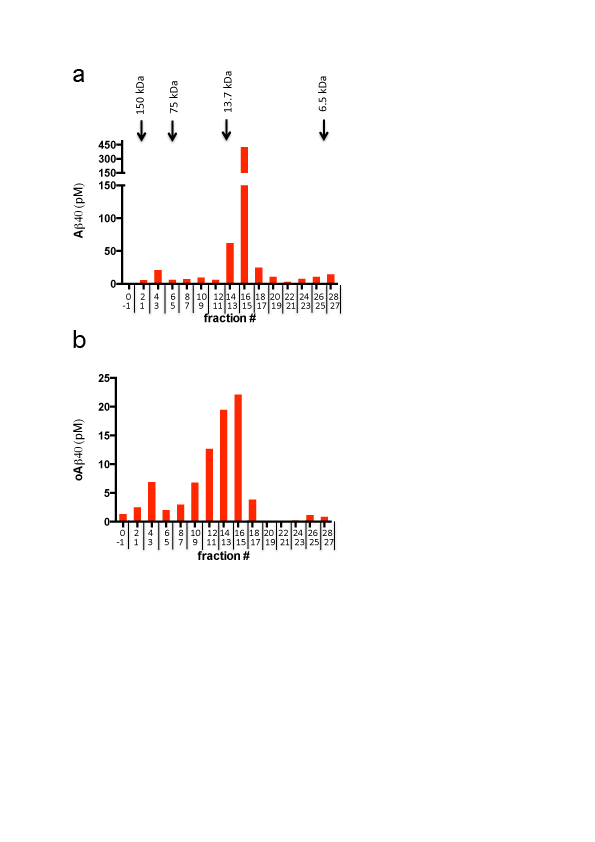

Supplement: Supplementary file 1 — Biochemical characterization of Aβ oligomers present in Tg conditioned media (TgCM). a Representative profile showing the amount of Aβ40 detected by ELISA in each fraction after separation of the different oligomeric species present in TgCM (red bars) by size-exclusion chromatography (SEC, Superdex 75 SEC columns). The molecular weight markers (kDa) that ran at the same conditions are indicated above (arrowheads). b Measurements of oligomeric Aβ within each SEC fraction show that most of oAβ is of low molecular weight (fractions 12–18).. A smaller peak of high-molecular weight species was also detected in TgCM (fractions 2–6 in both ELISAs). (TIF 1492 kb) [file 13024_2017_169_MOESM1_ESM.tif]

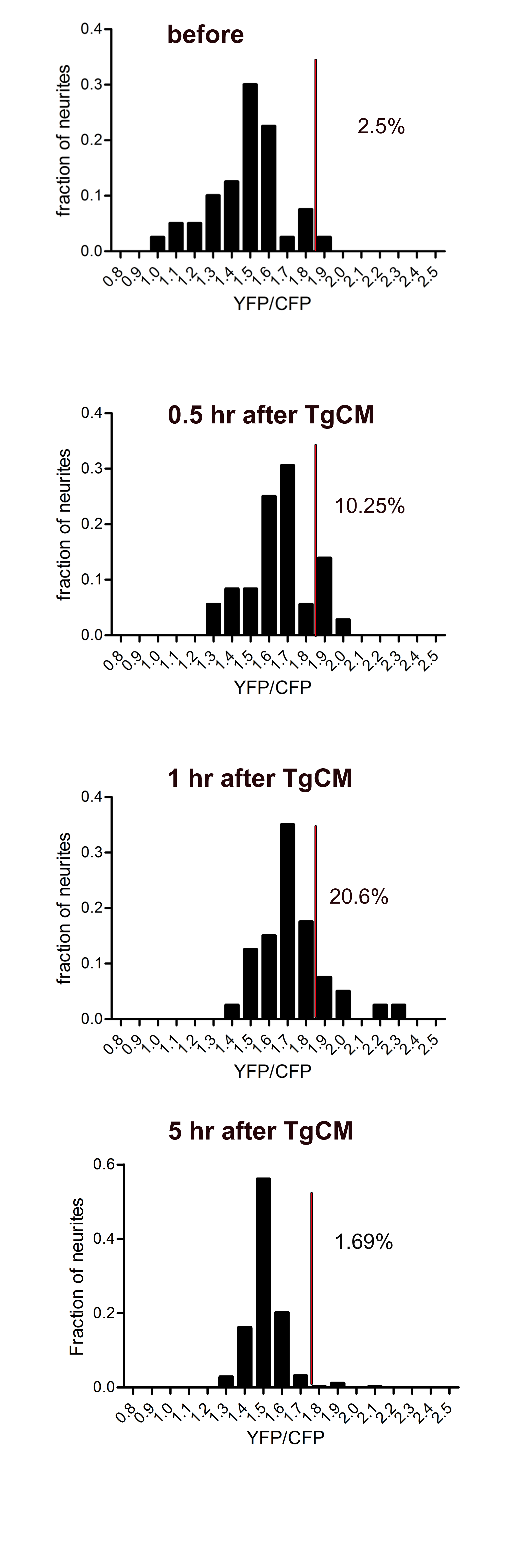

Supplement: Supplementary file 2 — Time course of oAβ-induced increase in resting calcium levels after acute exposure with TgCM on a healthy brain. C57BL/6 mice were injected with AAV-CBA-YC3.6 into the somatosensory cortex and acutely exposed to TgCM containing oAβ. The changes in YFP/CFP ratios in the neurites were measured at baseline before treatment and 30 min, 1 h and 5 h after topical application of TgCM. A progressive increase in the levels of resting calcium (YFP/CFP ratio and the actual calcium levels on the lower and upper x-axis, respectively) was detected that reached a peak after 1 h (20.6% of overloaded neurites), before returning to baseline levels after 5 h (1.7% overloaded neurites). (TIF 37844 kb) [file 13024_2017_169_MOESM2_ESM.tif]
